# Quality of life of children with neurodevelopmental disorders and their parents during the COVID-19 pandemic: a 1-year follow-up study

**DOI:** 10.1038/s41598-022-08273-2

**Published:** 2022-03-12

**Authors:** Riyo Ueda, Takashi Okada, Yosuke Kita, Masatoshi Ukezono, Miki Takada, Yuri Ozawa, Hisami Inoue, Mutsuki Shioda, Yoshimi Kono, Chika Kono, Yukiko Nakamura, Kaoru Amemiya, Ai Ito, Nobuko Sugiura, Yuichiro Matsuoka, Chinami Kaiga, Yasuko Shiraki, Masaya Kubota, Hiroshi Ozawa

**Affiliations:** 1 Department of Developmental Disorders, National Institute of Mental Health, National Center of Neurology and Psychiatry, 4-1-1 Ogawahigashi-Cho, Kodaira, Tokyo, 187-8553 Japan; 2 Mori Arinori Center for Higher Education and Global Mobility, Hitotsubashi University, Tokyo, Japan; 3 Cognitive Brain Research Unit (CBRU), Faculty of Medicine, University of Helsinki, Helsinki, Finland; 4 Department of Child Neurology, Shimada Ryoiku Medical Center Hachioji for Challenged Children, Tokyo, Japan

**Keywords:** Health care, Medical research

## Abstract

This study aimed to reveal changes in the quality of life (QOL) of children with neurodevelopmental disorders and their parents, and the interaction between their QOL and parental mental state during the coronavirus 2019 (COVID-19) pandemic. Eighty-nine school-aged children and parents participated in surveys in May 2020 (T1) and May 2021 (T2). The parents completed questionnaires that assessed their QOL, depression, parenting stress, and living conditions. Children’s temporary mood status was evaluated using the self-reported visual analog scale (VAS). Children’s QOL and VAS at T2 were higher than their QOL at T1. Parents’ QOL at T2 was lower than their QOL at T1. Severe parental depression at T1 had a synergistic effect on severe parenting stress and severe depressive state at T2. Additionally, children’s high QOL at T1 had a synergistic effect on low parenting stress and children’s high QOL at T2. Furthermore, children’s low VAS scores and parents’ low QOL at T2 were associated with deterioration of family economic status. Children and parents’ QOL changed during the prolonged COVID-19 pandemic. Improvement in children’s QOL was influenced by reduced maternal depressive symptoms. Public support for parental mental health is important to avoid decreasing QOL.

## Introduction

In December 2019, coronavirus 2019 (COVID-19) was first detected in China. The COVID-19 outbreak then quickly spread across the world to become a pandemic in March 2020. The COVID-19 pandemic has resulted in numerous changes in school-aged children’s daily lives, including wearing masks, social distancing recommendations, increased stay at home, and the cancelation of large-scale school events. Children experienced changes in their health and were more likely to gain body weight, spend less time doing physical activities, and spend more time online as compared to that in the pre-COVID-19 era^1^. According to the results of a systematic review, more than 30% of typically developing children (TDC) suffered from anxiety, depression, irritability, and inattention, and behavioral and psychological states of 79.4% were negatively affected during the COVID-19 pandemic^2^.

Particularly, children with neurodevelopmental disorders (NDDs) are more likely to experience mental and physical difficulties during a disaster, given the unpredictable changes around them and alterations to their routines in comparison with TDC^2–5^. In fact, the majority of children with autism spectrum disorder (ASD) had a higher probability of worsened behavioral symptoms during the pandemic than during the pre-COVID-19 era, and the need for professional support as perceived by parents was more for children with NDDs than for TDC^2,5^. Moreover, in a previous study, the prevalence of maternal depression increased especially in children with NDDs during the COVID-19 pandemic^6,7^, and maternal depressive state negatively interacted with changes in lifestyles and maladaptive behaviors of children with NDDs during the COVID-19 pandemic^1,3–5,8^. This negative interaction was observed between children with NDDs and their caregivers during ordinary times^9,10^ and previous natural disasters prior to the COVID-19 pandemic^11^, and was even worse during emergency situations.

In Japan, the total number of suicides in 2020 increased by 4.5% compared to the previous year, despite a decline in recent years. Particularly, the number of school-aged children (elementary school and junior and senior high school) who died by suicide in 2020 was 1.4 times higher than in 2019^12^. These results suggest that the COVID-19 pandemic in Japan, as well as in foreign countries, had deleterious effects on the mental health of the whole population, including children with NDDs and their parents. Since COVID-19 had a significant negative impact on children with NDDs and their parents, and children with NDDs find it difficult to adapt to lifestyle changes^2–5^, there is a rising concern that the mental health of children with NDDs and parents might have worsened during the early period of the COVID-19 pandemic.

In this study, we evaluated the quality of life (QOL) of children with NDDs and their parents in the early period of COVID-19 pandemic (May 2020; T1) and one year later (May 2021; T2) during the pandemic. The QOL describes an individual’s subjective perception of their position in life, as evidenced by their physical, psychological, and social functioning, and is often used to examine well-being and burden in children with NDDs and parents^8,13^. During the early period of the COVID-19 pandemic, previous studies revealed that higher QOL of children with NDDs and their parents was associated with less parental stress, parental depression and anxiety, and milder maladaptive behavior in children^8^. Caregivers of children with NDDs also reported a greater decrease in QOL than caregivers of TDC during the early period of the pandemic^13^. The change in the follow-up QOL (T2) of children with NDDs and from the early period of QOL (T1) is an important concern of this research.

This study aimed to examine the changes in the QOL of children and their parents over the first year of the pandemic as compared to the early period of COVID-19, and how T1 and T2 data of children's QOL and parents' mental health interacted with each other. We also explored possible factors affecting their QOL.

## Results

### Participant background

One hundred thirty-six people participated in the first survey, and their profiles were described in our previous study^8^. A total of 89 participants participated in the follow-up survey. The clinical backgrounds of the children with NDDs and their parents are shown in Table 1. Regarding the changes in children’s lifestyles, 17 children had increased exercise time (19.1%), 12 had lowered school attendance (13.5%), 53 had more game play time (59.6%), 44 had more Internet time (49.4%), and 12 children had acute onset obesity (13.5%) as compared to the pre-COVID-19 era. Regarding parents’ living conditions, 33 parents (questionnaire respondents) felt a greater burden on living expenses at T2 (37.1%) than before the COVID-19 era. Low-income households with welfare also increased from five households before the COVID-19 era (5.6%) to nine households at T2 (10.1%). Furthermore, 21 of the 80 fathers had less income at T2 (excluding single mother family; 26.3%) than during the pre-COVID-19 era, and 19 of 89 mothers had less income at T2 than during the pre-COVID-19 era (21.3%).

**Table 1:** Clinical background.

	Total
**Children**	
Male:female	70:19
Age M ± SD	11.6 ± 2.8
WISC/WAIS FSIQ M ± SD	85.1 ± 15.4
ADHD, N (%)	47 (52.8)
ASD, N (%)	49 (55.1)
SLD, N (%)	7 (7.9)
Increased exercise time, N (%)	17 (19.1)
Decreased school attendance, N (%)	12 (13.5)
Increased game play time, N (%)	53 (59.6)
Increased internet time, N (%)	44 (49.4)
Newly noticed obesity, N (%)	12 (13.5)
**Parent**	
Participants; mother:father:other than parents	84:3:2
Participants; age M ± SD	44.3 ± 5.3
Single-parent family (only mother), N (%)	9 (10.1)
Parental feelings of increased burden on living expenses, N (%)	33 (37.1)

(%) Data indicate the proportion of each characteristic in each group.

*N* number, *M* mean, *SD* standard deviation, *FSIQ* full-scale intellectual quotient, *ADHD* attention-deficit hyperactivity disorder, *ASD* autism spectrum disorder, *SLD* specific learning disorder.

### Assessment of the QOL of children and their parents

The results of the comparison between the T1 (May 2020) and T2 (May 2021) surveys are shown in Table 2. Parents’ scores at T2 on the averaged sub-scale values of WHO-QOL-BREF (0–100 scale) were significantly lower than their scores at T1. The children and parental domain scores of the Parenting Stress Index (PSI) at T2 were also lower than their scores at T1. Alternatively, there were no differences in the Center for Epidemiologic Studies Depression Scale (CES-D) scores, state scores, and trait scores of the State-Trait Anxiety Inventory (STAI) between the two groups. Regarding children, the averaged sub-scale scores of the Kiddo-KINDL and the VAS scores (children self-reported temporary mood status) at T2 were significantly higher than the original scores at T1. The total Child Behavior Checklist (CBCL) index at T2 was lower than the original index at T1.

**Table 2:** The results of paired t-tests of questionnaires.

	Follow-up (T2)	Original (T1)	*t *value	*p* value
Averaged sub-scale values of WHO-QOL-BREF, M (SD)	59.98 (9.65)	64.22 (10.50)	5.333	< 0.001*
CES-D, M (SD)	15.65 (11.52)	14.84 (9.22)	0.908	0.366
STAI, state, M (SD)	48.33(11.94)	48.61 (11.50)	0.286	0.775
STAI, trait, M (SD)	49.22 (12.43)	48.89 (11.42)	0.391	0.697
PSI, total domain, M (SD)	212.69 (44.23)	218.03 (40.68)	1.985	0.050
PSI, parent domain, M (SD)	111.94 (26.25)	115.89 (24.39)	2.862	0.005*
PSI, children domain	99.72 (24.29)	103.48 (19.45)	2.240	0.028*
Averaged sub-scale values of Kiddo-KINDL, M (SD)	74.23 (10.46)	71.69 (11.77)	2.768	0.007*
VAS, M (SD)	71.17 (20.03)	64.67 (22.61)	2.214	0.030*
CBCL, total index, M (SD)	65.70 (10.37)	67.84 (9.41)	2.986	0.004*

*M* mean, *SD* standard deviation, *WHO* World Health Organization, *QOL* quality of life, *CES-D* Center for Epidemiologic Studies Depression Scale, *STAI* State-Trait Anxiety Inventory, *PSI* Parenting Stress Index, *CBCL* Child Behavior Checklist.

T2 and T1 surveys were performed May 2021 and May 2020, respectively.

*p < 0.05.

**Figure 1:**
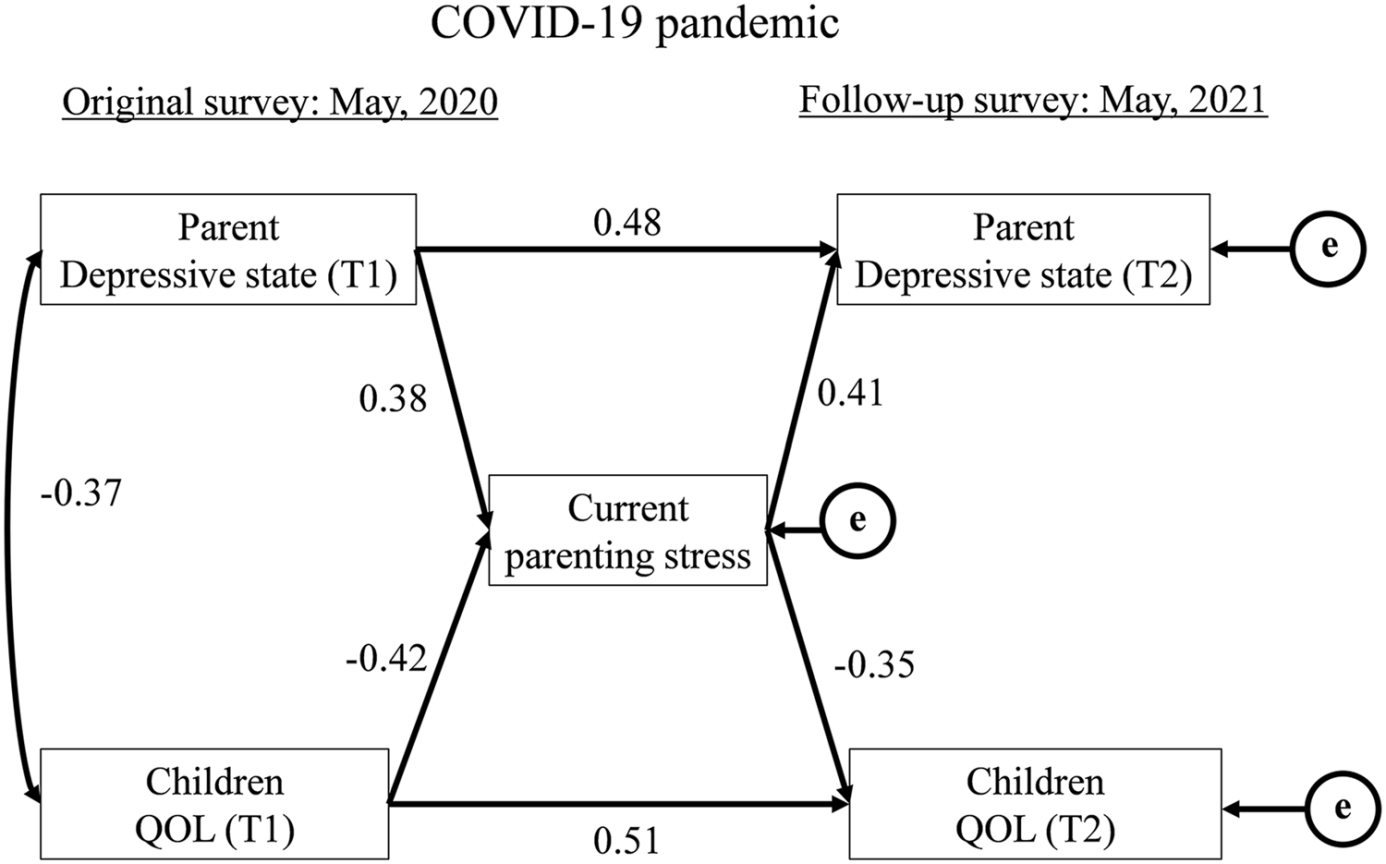
Interaction between maternal mental state and children’s QOL. The parents’ depressive state during the original survey (T1) and follow-up survey (T2) were evaluated using the Center for Epidemiologic Studies Depression Scale (CES-D) scores. Parenting stress at T2 was evaluated using the total domain of the Parenting Stress Index (PSI). The children’s QOL at T1 and T2 were assessed using the averaged sub-scale values of Kiddo-KINDL. The path analysis results showed that the model fit the data well (χ^2^ = 1.35 [3], *p* = 0.717, CMIN/DF = 0.450, GFI = 0.994, AGFI = 0.969, RMSEA < 0.001). A severe parental depressive state at T1 was directly associated with a severe depressive state at T2 (*β* = 0.48, *p* < 0.05), and was also associated with a higher total domain of PSI at T2 (*β* = 0.38, *p* < 0.05), which in turn was associated with a more severe parental depressive state at T2 (*β* = 0.41, *p* < 0.05)*.* Alternatively, children’s higher QOL at T1 was directly associated with their higher QOL at T2 (*β* = 0.51, *p* < 0.05), and their QOL at T1 had a negative relationship with parenting stress at T2 (*β* = -0.42, *p* < 0.05)*,* which in turn had an indirect negative relationship with children’s QOL at T2 (*β* = −0.35, *p* < 0.05)*.*

Figure 1 shows the interaction between children’s QOL and maternal mental state using the following parameters: QOL of children (T1 and T2), depressive state of parents (T1 and T2), and parenting stress (T2). The path analysis results showed that the model fit the data well (χ^2^ = 1.35 [3], *p* = 0.717, CMIN/DF = 0.450, GFI = 0.994, AGFI = 0.969, RMSEA < 0.001). Parents’ severe depressive state at T1 was directly associated with a more severe depressive state at T2 (*β* = 0.48, *p* < 0.05), and was also associated with a higher total domain of PSI at T2 (*β* = 0.38, *p* < 0.05), which in turn was associated with a more severe parental depressive state at T2 (*β* = 0.41, *p* < 0.05)*.* Alternatively, children’s higher QOL at T1 was directly associated with their higher QOL at T2 (*β* = 0.51, *p* < 0.05) and children’s QOL at T1 had a negative relationship with parenting stress at T2 (*β* = −0.42, *p* < 0.05)*,* which in turn had an indirect negative relationship with children’s QOL at T2 (*β* = −0.35, *p* < 0.05)*.*

Table 3 shows the changes in living-condition-related factors that significantly predicted the VAS at T2 in children and the parents’ QOL at T2. Stepwise multiple regression analysis for children’s VAS score at T2 also showed that a higher VAS score at T2 was associated with a higher VAS score at T1, lower children’s age, and parental feelings of decreased or unchanged burden of living expenses. In the parents’ QOL score at T2, parents’ lower QOL at T2 was associated with lower parental age, parents’ feeling of increased burden on living expenses, and parents’ lower QOL score at T1.

**Table 3:** Results of multiple regression analysis.

Parameter	*t* value	*β* coefficient	*p* value
**Parents’ QOL at T2**			
Intercept	0.14	–	0.892
Parents’ QOL at T1	9.85	0.707	< 0.001*
Parental feelings of increased burden on living expenses	2.08	−0.151	0.040*
Parents’ age	3.63	0.252	< 0.001*
*F* value	42.749		
*R*^2^	0.601		
**Children’s VAS at T2**			
Intercept	7.74	–	< 0.001*
Children’s VAS at T1	2.00	0.201	0.049*
Parental feeling increased of burden on living expenses	2.25	−0.225	0.027*
Children’s age	2.97	−0.298	0.004*
*F* value	6.492		
*R*^2^	0.190		

T2 and T1 surveys performed May, 2021, and May, 2020, respectively.

*QOL* quality of life, *VAS* visual analog scale.

*p < 0.05.

## Discussion

To our knowledge, this is the first study to reveal that the QOL of parents and children with NDDs changed, and that the children’s QOL and parental mental state interacted with each other during the prolonged COVID-19 pandemic. Namely, children’s higher QOL at T1 was associated with higher QOL at T2, which was affected by lowered parental depressive state at T1. Furthermore, the parents’ QOL at T2 was lower than their QOL at T1, and family economic status had a negative impact on parents’ QOL at T2 and temporary mood status at T2 of children with NDDs.

### The interaction between the QOL of children and depressive state of parents

Children’s QOL at T2 improved in comparison to their original QOL (T1), as demonstrated by path analysis. The interaction between children's QOL and parents' mental state was estimated not only at one point^8^, but was also maintained when the time axis was added in the follow-up study. Parents’ severer depressive state at T1 was associated with higher parenting stress at T2; higher parenting stress at T2 was also associated with severer parental depressive state at T2. Alternatively, children’s higher QOL at T1 was associated with lower parenting stress at T2. Lower parenting stress at T2 was also associated with children’s higher QOL at T2. These results indicated that there was a significant negative relationship between children’s QOL and parental depressive state during prolonged COVID-19 pandemic.

In a previous study, higher resilience among adolescents, which was defined as the ability to bounce back from social disadvantages or highly adverse conditions and has a positive relationship with QOL^14^, was associated with maternal parenting style (more maternal care, mothers’ autonomy-relevant parenting, and less maternal overprotection) after an earthquake^11^. Therefore, decreasing parental stress and maintained parenting skills are important to improve the QOL of children with NDDs during the prolonged COVID-19 pandemic.

Moreover, previous research showed the prevalence of maternal depression increased, especially in parents’ of children with NDDs, during the COVID-19 pandemic^6,7^, and parents of children with ASD had lower levels of resilience and coping skills against overall stress, not just parenting stress^7^. Additionally, maternal depressive state also showed a negative interaction directly with children’s unhealthy lifestyles and maladaptive behaviors during the COVID-19 pandemic^1,8^. In previous follow-up studies, psychiatric disorders, especially depression, were also related to reduced QOL in the adult population, not just parents with NDDs exposed to natural disasters^15,16^.

In this study, severe maternal depressive state during the early period of the COVID-19 pandemic (T1) was associated with lower QOL in children one year after the pandemic (T2). Furthermore, persistence of severe depressive state in parents might also affect the deterioration of parental QOL in the future. The results in Fig. 1 reveal that public support for parents’ mental state is important for the improvement or maintenance of QOL of children during the prolonged COVID-19 pandemic.

### Improvement of VAS of children in follow-up survey (T2)

Children’s VAS scores at T2 were higher than their original VAS scores (T1). The parental rating of QOL and children’s self-reported temporary mood status during the daytime improved in comparison with T1 data during the COVID-19 pandemic. Additionally, children’s higher temporary mood status at T2 was associated with their lower age and parental feelings of decreased or unchanged burden on living expenses. For parents who have children with ASD, caregiving’s impact on finances was associated with low parents' QOL in previous studies^17,18^. In this study, parents’ elevated feelings of burden on living expenses were associated with not only the deterioration of parents’ QOL but also with the lack of temporary mood status. Furthermore, it was revealed that health-related QOL of TDC in adolescents had a negative relationship with their age in previous study^19^, and hormonal changes in adolescents might lead to an impaired QOL^19^. Decreased VAS score in older children might be the result of biological changes in adolescence.

### Deterioration of QOL of parents during COVID-19 pandemic

Parents’ QOL at T2 was lower than their QOL at T1, and lower QOL at T2 was associated with an elevated feelings of burden on living expenses and lower age.

In a previous study, caregivers of children with ADHD and/or ASD reported lower QOL than caregivers of TDC, both before and during the pandemic^13^. In our study, parents’ QOL at T2 was lower than in the early period of the COVID-19 pandemic (T1). Parents of children with NDDs have been disproportionately affected by the pandemic. Parents’ QOL might have been affected by the long-term consequences of the COVID-19 pandemic, with reference to previous studies^20,21^. Six years after an earthquake and 30 years after food poisoning (polychlorinated biphenyls and dibenzofurans exposure), the QOL of adult survivors declined^20,21^. Although COVID-19 was qualitatively different from other disasters in that it had a long-term continuous impact on society, long-term deterioration of QOL in previous studies^20,21^ warned of similar changes during the COVID-19 pandemic.

Furthermore, previous studies have revealed that among adult survivors after the earthquake and gas explosion, low economic status or financial problems were associated with poorer QOL^22–24^, and well-targeted post-disaster financial/material aid and social support had to be considered as means for improving the long-term QOL outcomes of disaster survivors^24^. Similarly, our study revealed that parents’ decreased QOL was influenced by their feelings of increased burden on living expenses. Therefore, long-term deterioration of parents’ QOL should be avoided or reduced by financial support during the prolonged pandemic. Moreover, previous studies demonstrated that younger adults were more likely to have lower QOL and more severe mental problems than older adults during the COVID-19 pandemic^25,26^. Similar to other studies^25,26^, younger parents were more likely to have a lower QOL than older parents for biological reasons in our study.

This study had several limitations. First, the number of participants analyzed in the study was small, and the diagnoses of NDDs varied. However, all participants were from families in Hachioji and its neighboring cities, which are commuter towns. The rate of receiving welfare in Hachioji is similar to that of the national average. The families had relatively homogeneous lifestyle patterns. It was estimated that traveling between the city center and the suburbs caused an increase in the number of COVID-19-infected people in these cities, as in most parts of Japan. Second, it is unclear whether the QOL of parents and children was lower during COVID-19 than that before COVID-19, because the QOL of parents and children with NDDs is always significantly lower than that of the general population^27–29^. Third, the VAS was conducted by the children themselves, which might have resulted in inadequate control of the experimental environment, especially in younger participants’ cases. However, the VAS has been widely used for the subjective evaluation of pain, anxiety, well-being, etc. including in children over the age of six years and with NDDs in previous studies. In our study, the VAS was used because it directly reflected the children's own mental status^30–32^. Fourth, information about low-income households and the alteration of income from before and during the COVID-19 pandemic lacked accuracy, since these data were parental self-managed reports. However, it was significant that parents and children’s QOL changed as the COVID-19 pandemic prolonged, and the factors affecting QOL could be identified.

In conclusion, this study discussed the QOL of children with NDDs and their parents, comparing the early period (T1) and one year after (T2) the COVID-19 pandemic. Children’s QOL and VAS scores at T2 were higher than their original scores (T1). Alternatively, parents’ QOL at T2 was lower than their QOL at T1. There was a significant interaction between changes in parental mental state and QOL in children. Improvements in children's QOL were indirectly associated with improvements in maternal depressive symptoms, via mothers' current parenting stress. To improve the QOL of children, it is essential to not only provide public support for children, but also expand mental and financial support to parents. It might be expected that the QOL and mental health of children with NDDs and their parents will continue to change in the future, while affecting each other and at the same time being influenced by social conditions and public support. This hypothesis needs to be clarified in further follow-up studies.

## Methods

### Data collection

In this prospective study, we enrolled 89 school-aged children with NDDs and their parents (84 mothers [94.4%], three fathers [3.4%], one grandmother [1.1%], and one adult sister [1.1%]) at the Shimada Ryoiku Center Hachioji in Hachioji City, a regional core outpatient clinic where children receive medical examinations, rehabilitation, and psychotherapy.

Hachioji is located in the western suburbs of Tokyo. It is a commuter town with a population of 580,000 (population density 3093 km^2^). Low-income households in Hachioji requiring welfare as per the Public Assistance Act accounted for 1.75% of all households, as compared to the 1.68% nationwide average^33^. Participant inclusion criteria were (1) children with NDDs (attention-deficit hyperactivity disorder [ADHD], ASD, specific learning disorders [SLD], tic disorders, or other NDDs), as classified by Diagnostic and Statistical Manual of mental disorders (DSM-5), and (2) school-aged children (from 6 to 18 years old). The children’s NDD diagnoses were confirmed by the attending physician. All children and their parents were evaluated twice, in May 2020 and May 2021.

The first state of emergency was declared in May 2020 during the period of the T1 survey, and a third state of emergency was declared in May 2021 during the period of the T2 survey. School attendance was suspended in May 2020; however, it remained regular in May 2021. During these periods, there were six persons infected with COVID-19 in May 2020 (a 5.2% rate of averaged positivity by polymerase chain reaction [PCR] assay) and 645 persons in May 2021 (12.0% rate of averaged positivity by PCR assay). The double vaccination rate was 0.54%, on May 31, 2021, in Japan.

### Assessment tools

One caregiver from each family completed the following questionnaires at each time point: (1) an assessment of the parent’s clinical status, the Japanese version of the State-Trait Anxiety Inventory (STAI)^34^, the Center for Epidemiologic Studies Depression Scale (CES-D)^35,36^, the Parenting Stress Index (PSI)^37,38^, and the World Health Organization Quality of Life-BREF (WHOQOL-BREF)^39^; (2) an assessment of the children’s clinical status, the Child Behavior Checklist (CBCL)^40^ and the KINDL^R^ to measure health-related QOL^41,42^. The questionnaires were completed by the same caregiver at the T1 and T2 surveys. The score calculation method of the Kiddo-KINDL^R^ and WHOQOL-BREF was the same as in our previous study^8^. The average values in the of the four sub-scales in the Kiddo-KINDL^R^ (physical well-being, emotional well-being, self-esteem, and family), excluding social contact and school sub-scales, were calculated to evaluate the children’s QOL, since social contact and school sub-scales were not available in the T1 survey. Parents were also assessed for changes in living conditions during the COVID-19 pandemic based on parents’ perceptions in the follow-up survey, including exercise at home, school attendance frequency, amount of time spent using the Internet and playing games, acute onset obesity, changes in parental feelings of burden on living expenses, and changes in the level of income.

Children were also asked to complete the VAS as a follow-up with their subjective temporary mood status in terms of percentages of the best imaginable state. The VAS includes a graph scale on a horizontal line with endpoint 0 (the worst mood status imaginable, the picture of a crying face), mid-point 50 (intermediate mood status, the picture of a neutral face), and opposite endpoint 100 (the best mood status imaginable, the picture of a smiling face). The face scale was added to the VAS to increase non-verbal explanations for children with different verbal abilities^43^. The VAS was performed by the children themselves, using a common test form (Supplementary Fig. S1) when the child was calm. Before the survey, dedicated staff instructed the children and their parents on how to explain the test. The same parent accompanied during the VAS examination at T1 and T2 surveys. In addition, children with moderate or profound intellectual disabilities were excluded (because it was considered difficult for children to correctly understand the VAS questions).

Children’s intellectual quotient scores were confirmed by the attending physician and were checked within one year before the start of the study. Wechsler Adult Intelligence Scale-Fourth Edition (WAIS-IV) was applied to children 17 years and older, and Wechsler Intelligence Scale for Children-Fourth Edition (WISC-IV) was applied to 16-year-old children or less. Questionnaires were collected by mail.

### Statistical analysis

First, paired t-tests between T1 and T2 surveys on the Kiddo-KINDL^R^, total indexes of CBCL, WHOQOL-BREF, total domain, child domain, and parent domain scores of PSI, state-anxiety scores, and trait anxiety scores of the STAI, and CES-D scores were performed.

Second, path analysis was conducted to assess the relationship between children’s QOL at T1 and T2, CES-D at T1 and T2, and parents’ total domain scores of PSI at T2.

Third, multiple regression analyses were used to identify changes in living-condition-related factors during the prolonged COVID-19 pandemic, age of parents, and QOL of parents at T1 that significantly affected parents’ QOL at T2. Additionally, multiple regression analyses were used to identify changes in living-condition-related factors during the prolonged COVID-19 pandemic, age of children, and children’s VAS at T1 that significantly affected their VAS at T2. Significant explanatory variables were selected based on Akaike information and Bayesian information criteria. Significance was set at *p* < 0.05.

Statistical analyses for Tables 1, 2 and 3 were generated using JMP software, version 9.0.3 Copyright © 2010 SAS Institute Inc. The SAS and all other SAS Institute Inc. product or service names are registered trademarks of SAS Institute Inc., Cary, NC, USA. Statistical analyses for Fig. 1 were conducted using IBM SPSS Amos 19 (SPSS Japan Inc., Tokyo, Japan).

### Ethical approval and informed consent

This study followed the guidelines of the Declaration of Helsinki and was approved by the institutional review board of the Shimada Ryoiku Medical Center Hachioji for Challenged Children (Shimahachi-2001). For all participants, dedicated staff explained the study while maintaining social distance in a well-ventilated large room, and the parents agreed to participate and provided their written informed consent.

## Supplementary Information

Supplementary Figure 1.

## Data Availability

Data are available upon reasonable request.
