# Supplementary material for: Quality of life of children with neurodevelopmental disorders and their parents during the COVID-19 pandemic: a 1-year follow-up study
Source: Sci Rep. 2022 Mar 12;12:4298. doi: 10.1038/s41598-022-08273-2 (PMC8918332; doi:10.1038/s41598-022-08273-2)
Supplement: Supplementary file 1 — Supplementary Figure 1. [file 41598_2022_8273_MOESM1_ESM.pptx]

## Slide 1
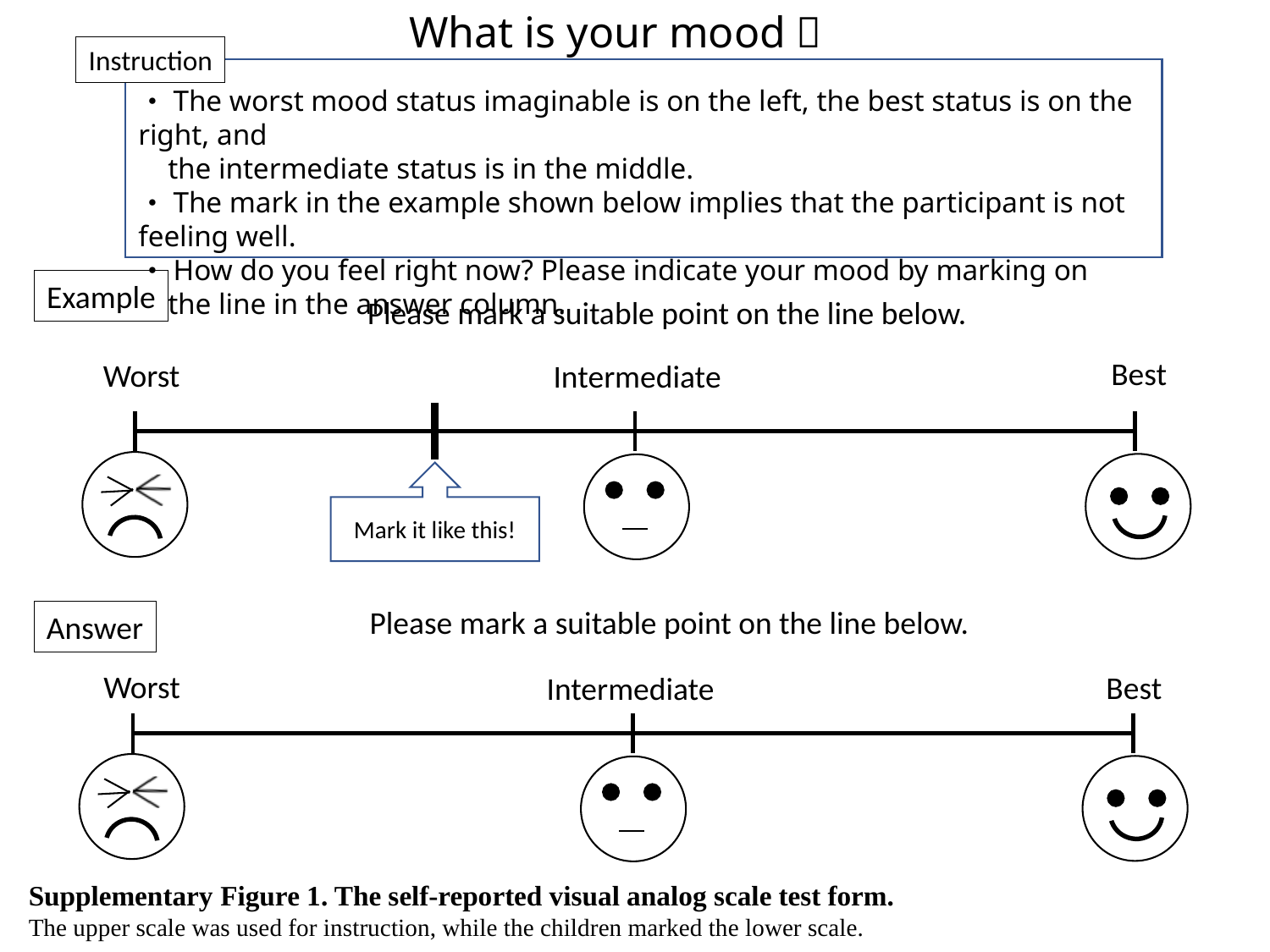

# What is your mood？
Instruction
・The worst mood status imaginable is on the left, the best status is on the right, and
 the intermediate status is in the middle.
・The mark in the example shown below implies that the participant is not feeling well.
・How do you feel right now? Please indicate your mood by marking on
 the line in the answer column.
Example
Please mark a suitable point on the line below.
Best
Worst
Intermediate
Mark it like this!
Please mark a suitable point on the line below.
Answer
Worst
Best
Intermediate
Supplementary Figure 1. The self-reported visual analog scale test form.
The upper scale was used for instruction, while the children marked the lower scale.
